# Effects of Daily Kelp (*Laminaria japonica*) Intake on Body Composition, Serum Lipid Levels, and Thyroid Hormone Levels in Healthy Japanese Adults: A Randomized, Double-Blind Study

**DOI:** 10.3390/md19070352

**Published:** 2021-06-22

**Authors:** Seiichiro Aoe, Chiemi Yamanaka, Hirofumi Ohtoshi, Fumiko Nakamura, Suguru Fujiwara

**Affiliations:** 1 Department of Food Science, Otsuma Women’s University, Chiyoda-ku, Tokyo 102-8357, Japan; 2 The Institute of Human Culture Studies, Otsuma Women’s University, Chiyoda-ku, Tokyo 102-8357, Japan; chiemiy@gmail.com; 3 Maruyanagi Foods Inc., Kobe, Hyogo 658-0044, Japan; h-otoshi@maruyanagi.co.jp; 4 CPCC Company Limited, Chiyoda-ku, Tokyo 101-0047, Japan; f.nakamura@cpcc.co.jp (F.N.); s.fujiwara@cpcc.co.jp (S.F.)

**Keywords:** body fat percentage, alginate, kelp, iodine, thyroid hormone

## Abstract

To investigate whether supplementation with iodine-reduced kelp (*Laminaria japonica*) powder decreases body fat composition in overweight Japanese subjects, a randomized, double-blind, placebo-controlled intervention study was conducted in 50 Japanese subjects with body mass index (BMI) ≥25 and <30 kg/m^2^. Subjects were randomly assigned to consume thirty tablets/d (10 tablets orally, 3 times/d) containing either iodine-reduced kelp powder (test, 6 g kelp powder corresponding to 3 g alginate/d) or kelp-free powder (placebo) for 8 weeks. Anthropometric measurements, blood lipids, and serum thyroid hormone levels were obtained before and after the trial. Body fat percentage was significantly decreased in male subjects from the test group compared with the placebo group. The same tendency was observed for body weight (*p* = 0.065) and BMI (*p* = 0.072) in male subjects. No significant changes in anthropometric measurements or visceral fat area were observed in female subjects. Serum thyroid hormone concentrations did not increase after 1.03 mg/d of iodine supplementation through kelp intake. The intake of iodine-reduced kelp powder led to significant and safe reductions in body fat percentage in overweight male subjects. The consumption of kelp high in alginate may contribute to preventing obesity without influencing thyroid function in Japanese subjects with a relatively high intake of iodine from seaweed.

## 1. Introduction

Obesity is a severe health problem and a key risk factor for other diseases, including type 2 diabetes, hypertension, dyslipidemia, and coronary heart disease [1]. Metabolic syndrome (MS) is a cluster of symptoms, including abdominal obesity, dyslipidemia, hypertension, and glucose intolerance [2]. Improving nutrient intake is a key factor in reducing the risk of MS. Dietary fiber is one of the candidates for improving nutrient intake: epidemiological evidence has shown a negative relationship between dietary fiber intake and the incidence of type 2 diabetes [3,4], hypertension, dyslipidemia [5], and coronary heart disease [6]. However, the average daily fiber intake in the Japanese population has been low for many years (<15 g/d) [7].

Seaweed, especially kelp (*Laminaria*), is a good source of dietary fiber; the soluble fibers consist of alginates, laminarin (β1–3, β1-6-glucan), and fucoidan, whereas the insoluble fiber is essentially cellulose [8,9]. Seaweed is an important dietary component for people in Japan: it is served in approximately 21% of meals [10]; however, the daily median consumption per person was reported to be 1.2 g/d, and the contribution of dietary fiber intake from seaweed to total dietary fiber intake was only 0.4 g/d [7]. These results suggest that the consumption of dietary fiber-rich seaweed could be increased in the Japanese diet. The major seaweeds consumed include Wakame (*Undaria*), Nori (*Porphyra*), Konbu (*Laminaria*), and Hijiki (*Hizikia*).

The consumption of seaweeds has been reported to affect both health status and chronic disease: several studies have reported a relationship between seaweed intake and risk reduction in cardiovascular disease [11], metabolic syndrome [12], and type 2 diabetes [13]. In addition, effects of seaweed and seaweed extract intake on digestive tract health [14], bone health [15], and cancer prevention [11], as well as antiviral properties [16], have been reported; however, there are few reliable human studies.

Seaweed also contains bioactive compounds with high antioxidant capacity, such as carotenoids and polyphenols [17,18]. Reports have shown that fucoxanthin, from different types of algae, has anti-inflammatory and anti-obesity effects [19,20].

The effects of whole seaweed meals and seaweed extract on obesity have been widely studied in both animal and human intervention studies. In diet-induced obese mice, feeding Wakame (*Undaria*) produced positive effects on body weight gain and glucose metabolism [21]. Another investigation reported body weight reduction and reduced serum total cholesterol and triacylglycerol levels in mice fed a high-fat diet containing extracts from red seaweed (*Gelidium amansii*) [22].

There are very few reports on the effects of kelp intake on body weight loss: only one report showed a significant reduction in serum cholesterol using Japanese subjects given 5 g of seaweed powder daily [23]; however, the control and subject information were not completely described.

Many species of red, green, and brown algae (seaweed) are used in Japanese meals [24]. One of the three most popular seaweed products in Japan is kelp, which contains 2353 μg/g of iodine [25,26]. Since several grams of kelp powder supplementation exceeds the tolerable upper intake level (3000 μg/d in adults) of iodine [27], it is difficult to conduct intervention studies on body weight loss using kelp powder. Approximately 80% of the iodine in kelp is released after boiling [28]; therefore, we conducted an intervention study using iodine-reduced kelp powder produced through a boiling process.

The purpose of this study was to confirm whether supplementation with boiled kelp powder causes body weight and body fat loss in overweight Japanese subjects (BMI 25–30 kg/m^2^). Blood lipids and thyroid hormone levels were also examined. To our knowledge, this is the first randomized, double-blind, placebo-controlled trial to evaluate the effects of iodine-reduced kelp in Japanese participants.

## 2. Results

### 2.1. Subject Characteristics

**Figure 1:**
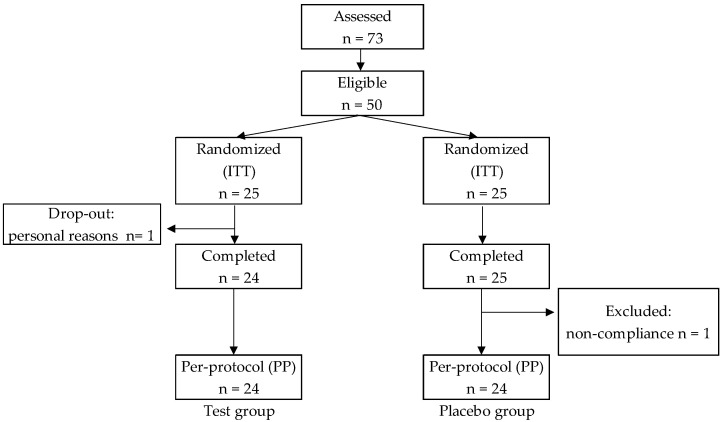
Flow of subjects through the 8-week intervention study (ITT: intention to treat).

A total of 50 subjects were shown to be eligible to participate in the study. One subject dropped out for personal reasons. All other subjects completed the trial. Two subjects were excluded: one dropped out for personal reasons in the test group, and the other was excluded for non-compliance in the placebo group (Figure 1). Table 1 summarizes the baseline characteristics of the subjects. The two groups were similar in age, body weight, BMI, body fat percentage, and visceral fat area. Blood and urine parameters did not differ significantly between the groups after the intervention period (data not shown). One subject in the test group had allergic symptoms, but not kelp allergy symptoms. Other adverse events corresponding to the intake of the kelp supplement, such as gastrointestinal problems, were not observed during the trial. Statistical analyses were performed for the subjects in the placebo group (n = 24) and test group (n = 24) in the per-protocol set analysis.

### 2.2. Daily Energy and Nutrient Intakes

Daily energy and nutrient intakes during the experimental period are summarized in Table 2. There were no significant differences between the calorie intakes of the test and placebo groups during the trial. There were also no significant differences between the nutrient intakes (protein, fat, carbohydrate, cholesterol, total dietary fiber, calcium, and iodine) of the test and placebo groups. The average daily iodine intake was 0.57 mg/d at week 0 and 0.76 mg/d at week 8; therefore, subjects did not exceed the tolerable upper intake level of 3 mg/d of iodine (Dietary Reference Intakes for Japanese (2020)) through the intake of supplements (1.03 mg/d).

### 2.3. Anthropometric Measurements and Visceral Fat Area

**Figure 2:**
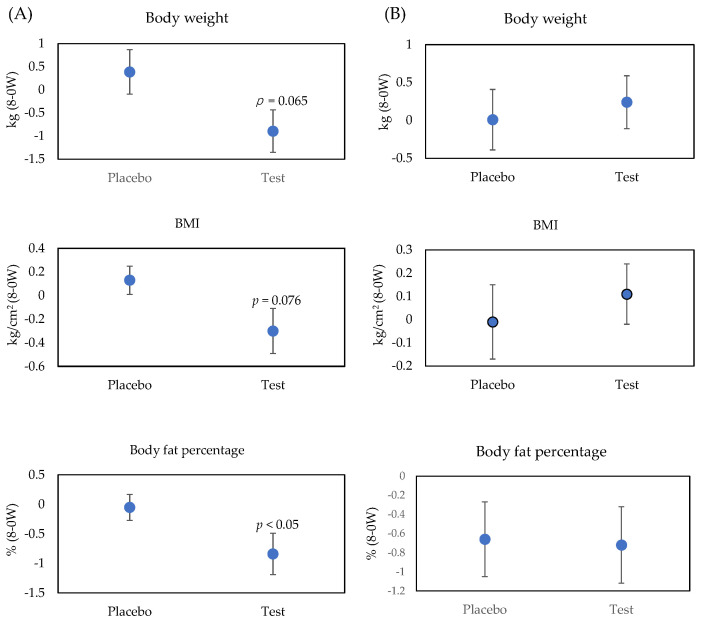
Effects of boiled kelp powder on body weight, BMI, and body fat percentage (bars represent means and SE): (**A**) male, (**B**) female.

Changes in body weight, BMI, and body fat percentage after 8-week consumption of boiled kelp powder are shown in Figure 2. Anthropometric measurements and visceral fat area are shown in Table S1. These measurements were analyzed after stratification by gender. In males, the decrease in the body fat percentage was significantly greater in the test group compared with the placebo group. The same tendency was observed in body weight (*p* = 0.065) and BMI (*p* = 0.072). No significant differences between the placebo group and the test group in visceral fat area or systolic and diastolic blood pressure were observed in males. In females, body weight and BMI showed a small change in the placebo group (0.01 kg and −0.01 kg/cm^2^), whereas these parameters were increased in the test group (0.24 kg and 0.11 kg/cm^2^); however, these differences were not statistically significant (*p* = 0.665 and 0.587). In females, no significant changes were observed in body fat percentage, visceral fat area, or either of the blood pressure measurements.

### 2.4. Serum Lipid Values

Serum lipid values are shown in Table 3. Serum lipids were analyzed in all subjects: hyperlipidemia and non-hyperlipidemia subjects (total cholesterol <5.172 mmol/L, LDL cholesterol <3.879 mmol/L, and triglyceride <1.693 mmol/L). In the overall analysis, no significant differences were observed in serum lipid levels. In the non-hyperlipidemia subjects, LDL cholesterol levels were significantly lower in the test group at 8 weeks when compared with the placebo group: LDL cholesterol levels were reduced in the test group (−0.17 mmol/L), whereas they increased in the placebo group (+0.19 mmol, *p* = 0.063). The same tendency was observed for total cholesterol levels at 8 weeks in non-hyperlipidemia subjects (*p* = 0.05). No significant differences in HDL cholesterol levels were observed between the groups in non-hyperlipidemia subjects. No significant differences in serum lipid levels were observed in hyperlipidemia subjects because of the wide variation.

### 2.5. Serum Thyroid Hormones

Serum thyroid hormone concentrations are shown in Table 4. Group differences and changes during the experimental period were not observed. Serum thyroid hormone concentrations (TSH, FT3, and TF4) did not increase after 1.03 mg/d of iodine supplementation through kelp intake.

## 3. Discussion

In a parallel, double-blind, placebo-controlled study, we randomly assigned 48 overweight (BMI 25–30 kg/m^2^) subjects to either a placebo supplement or a kelp supplement (6 g kelp powder/d, 3.3 g alginate/d) group. No differences in the changes in body fat percentage and visceral fat area (VFA) between groups were shown in the per-protocol (PP) analysis; however, in the male subjects, there was a significant body fat percentage loss within the test group compared with the placebo group. In the PP analysis, serum concentrations of total, LDL, and HDL cholesterol and triglyceride concentrations did not differ between the groups; however, after sub-analysis in non-hyperlipidemic subjects, serum LDL cholesterol concentration was significantly different at 8 weeks between the test and placebo groups.

We hypothesized that supplementation with a boiled kelp powder rich in alginate could enhance body fat loss and improve body composition in overweight Japanese subjects. A large reduction in body fat percentage was previously reported after alginate supplementation (45 g/d for 12 weeks) [29]; however, other alginate supplementation studies have reported conflicting results [30]. To our knowledge, this is the first randomized, double-blind, placebo-controlled trial to evaluate the effects of boiled kelp powder in overweight Japanese subjects (BMI 25–30 kg/m^2^).

In this study, body fat loss after kelp intake was observed only in male subjects. Men and women exhibit significant differences in obesity and cardiovascular disease [31]. Men generally have a greater incidence of obesity due to increased visceral adipose tissue; women tend to have increased fat mass proportional to their body weight due to increased subcutaneous adipose tissue. Generally, Japanese women have much lower rates of MS (14.3% in women, 25% in men) [32]. A previous study used a higher dose of seaweed in female subjects but did not report any changes in weight; however, a decreased waist circumference among female subjects treated with the higher dose of seaweed was observed and was suggested to be modulated by estrogen metabolism [33]. Our previous study on cereal fiber intake showed a reduction in visceral fat weight when the visceral fat area was more than 100 cm^2^ [34]. In this study, there were seven subjects (5 male and 2 female) in the test group and only three (2 male and 1 female) in the placebo group with a visceral fat area more than 100 cm^2^, and this is suggested to be the reason why we did not observe a reduction in visceral fat in all subjects and body fat loss in female subjects. Further studies are needed to elucidate the effect of kelp intake on visceral fat reduction in abdominal obesity subjects.

Dietary fiber has been reported to prolong gastric emptying time, thereby enhancing satiety and resulting in a reduction in food intake [35]. Seaweed is a good source of dietary fiber, and it is reported that alginate isolated from seaweed may attenuate appetite and associated markers [36]. In our study, the daily energy intake at 0 and 8 weeks did not differ between the groups, suggesting that the reduction in body fat percentage in men was not due to prolonged gastric emptying time.

Alginate is a typical viscous soluble fiber, which can inhibit the absorption of carbohydrate and lipid from the gut [37]. Repeated reductions in carbohydrate and lipid absorption rate by alginate consumption might protect against body fat accumulation. We hypothesized that this could be one possible explanation for the reduced body fat percentage observed in the kelp group compared with the placebo group.

We also investigated whether the intake of alginate from kelp would lead to improvements in cholesterol metabolism, which have been proposed with similar viscous soluble fibers [38]. The hypocholesterolemic effect of dietary fiber has been attributed to its ability to inhibit the intestinal absorption of bile acids and neutral steroids, resulting in increased fecal bile acids and neutral steroid excretions [39]. It is speculated that alginate in kelp increases the excretion of bile acids and neutral steroids, suggesting a causative role in the lowering of serum cholesterol concentrations.

It has been estimated that the average Japanese iodine intake is 1–3 mg/d [40]. Kelp contains iodine, and the additional iodine supplementation in our study was 1 mg/d, which did not increase serum TSH levels. It is unlikely that the results observed in our study could be attributed to the iodine supplementation since a previous study reported that intake of 0.5 mg/d iodine from seaweed produced only minor transient changes in serum TSH [41]. A previous study reported that seven weeks of 5 g/d seaweed supplementation in American women was associated with a small but statistically significant increase in TSH [41]. Therefore, our result may be a phenomenon peculiar to Japanese subjects with relatively high intake of iodine from seaweed. Further studies are needed to clarify the influence of iodine intake from kelp.

Ethnic differences in lipid and glucose metabolism are mainly caused by frequency differences in genetic polymorphism. This difference results in the rapid increase in obesity and type 2 diabetes among mongoloids exposed to a westernized diet. In addition, the results of intervention studies may also differ. Some Asian countries, such as Korea, Japan, and parts of China, consume the greatest proportion of seaweed; therefore, the results of seaweed intervention studies may differ from those in Western countries due to differences in iodine tolerance and microbiota fermentation, as well as genetic differences. Kelp intervention studies are rarely reported in African and European countries; therefore, it is difficult to extrapolate our data for other races. Further studies are needed to elucidate the effect of kelp intake on the anti-obesity effect in all races.

A major limitation of the study was the small sample size: a larger study is planned for the future. A second limitation was the study design: there were quite a few subjects with abdominal obesity in spite of the BMI inclusion criteria of 25–30 kg/m^2^. Future studies are needed to elucidate the effect of kelp on visceral fat area loss in subjects with a VFA larger than 100 cm^2^.

## 4. Materials and Methods

### 4.1. Subjects

The study was designed to conform with the regulations of the Institutional Review Board of Chiyoda Paramedical Care Clinic according to the ethical guidelines for medical and health research involving human subjects [42] (IRB No. 15000088) and the Declaration of Helsinki. All subjects gave written informed consent before entering the study. The primary purpose of this study was to estimate the effects of boiled kelp powder on body fat reduction in subjects with high BMI.

The inclusion criteria were:

1. Aged 20–59 years;
2. BMI 25–30 kg/m^2^.

The exclusion criteria were:

1. Having a history of a severe circulatory, respiratory, digestive, urinary system, endocrine, or blood disease;
2. Receiving therapeutic medication at the time of the agreement of informed consent;
3. Having a food or drug allergy;
4. Will not eat kelp;
5. Regular consumption of foods for specified health use, foods with functional claims, supplements, and health foods rich in dietary fiber;
6. Refraining from or restricted from iodine-rich foods;
7. Having an excessive alcohol intake (more than 30 g/d of alcohol);
8. Having extremely irregular eating habits or irregular rhythms of life such as shift workers and late-night workers;
9. Participating in another study involving the intake of drugs or food within one month before screening tests or planning to participate in another study involving the intake of drugs or food after the agreement of informed consent;
10. Any male who donated 400 mL of whole blood 3 months before the start of the trial;
11. Any female who donated 400 mL of whole blood 4 months before the start of the trial.

Fifty subjects were randomly assigned into each trial group (boiled kelp powder group and placebo group), stratified by visceral fat area, BMI, age, sex, and serum LDL cholesterol levels. All subjects were Japanese men and women. The study was designed as a double-blind, controlled, randomized trial. Subjects consumed ten test tablets three times a day (30 tablets daily) at each meal for 8 weeks. The study period was determined to be 8 weeks, which is an intermediate period of a previous report (4 to 12 weeks) [43].

### 4.2. Test Supplements

The components of the placebo and test tablets are listed in Table 5. Kelp (Japanese kelp; *Laminaria japonica*, Hokkaido Fisheries Corporative Associations, Hokkaido, Japan) was boiled for 60 min to remove iodine before being dried and powdered. The total dietary fiber content in boiled kelp powder was analyzed using the method of Prosky et al. (AOAC 991.43) [44]. The alginate content was analyzed by a colorimetric method, using carbazole reagent. The iodine content was analyzed by GC-ECD. The above analyses were performed by a commercial laboratory (Japan Food Research Laboratories, Tokyo, Japan). The content of alginate in the intact kelp powder was 26.8 g/100 g, whereas that in boiled kelp powder was 55.6 g/100 g. The weight of one test tablet was 500 mg, and it contained 0.11 g total dietary fiber, 34.3 μg iodine, and 109.3 mg alginate. The placebo and test tablets had the same appearance and flavor.

### 4.3. Study Design and Intervention

Both the subjects and researchers were unaware of the assignment of tablets. Subjects consumed ten tablets (placebo or test tablets) three times a day at their three main daily meals (breakfast, lunch, and dinner) for 8 weeks (total 30 tablets daily). The subjects in the test group had a daily alginate and iodine consumption of 3.28 g (30 × 0.1093 g) and 1.03 mg (30 × 0.0343 mg), respectively. The dosage of alginate was determined according to a previous report using 2.8 g/d of alginate beverage in obese and overweight women [45]. Subjects ate self-selected foods, abstained from excessive eating and drinking, and otherwise maintained the same eating habits before and throughout the trial. During the trial, subjects completed daily intake records specifying the amount and time of each experimental tablet intake and provided a three-day record of their diet and daily activity at baseline (before intervention) and during the 8th week of the trial (final week). Food records were analyzed using the Standard Tables of Food Composition in Japan 2015, Seventh Revised Version [46] and the software program Excel Eiyokun Version 8 (Kenpakusha, Tokyo, Japan). In Japanese food, most iodine intake comes from Japanese soup which contains the kelp extract “dashi”. Since the amount of iodine varies significantly depending on the type of soup stock and miso used, the iodine intake was uniformly calculated using Japanese granule soup stock and light-colored spicy miso. If subjects consumed less than 30 tablets per day or their recorded intake diary did not conform to the protocol for more than 85% of the trial, their data were excluded. During the initial and final weeks of the experimental period, anthropometric measurements (body weight, height, body fat percentage, and visceral fat area) were performed, and blood samples were collected from a forearm vein and analyzed.

### 4.4. Clinical Analyses

Body weight and body fat percentage was measured using an InBody570 body composition analyzer (InBody Japan, Inc., Tokyo, Japan). Visceral fat areas were measured with a DUAL SCAN (HDS-2000, OMRON HEALTHCARE Co., Ltd., Kyoto, Japan). Areas of visceral and total fat were calculated for each slice. Body weight and height were assessed, and BMI was calculated. Blood and urine samples were obtained after an overnight fast by a commercial laboratory (LSI Medience Corporation, Tokyo, Japan). Serum thyroid hormone levels (TSH, FT3, FT4) were measured by chemiluminescent immunoassays (Chemilumi-ACS-TSH, Chemilumi-ACS-FT3, Chemilumi-ACS-FT4, respectively; Roche Diagnostics, Indianapolis, IN, USA). Serum thyroid hormone levels are markers of thyroid function.

### 4.5. Statistical Analysis

Sample sizes were calculated from previous studies [47]. More than twenty subjects were required per group (type I error (α) = 0.05, 1–β = 0.80). Data normality was checked using a quantile–quantile plot. Bartlett’s test was used to measure the homogeneity of variances. Data were analyzed using JMP (Version 14.0, SAS Institute Inc., Cary, NC, USA). Unpaired *t*-test, Welch’s *t*-test, or Wilcoxon rank sum test was used to evaluate: (1) changes from baseline and differences between the changes in the test and placebo groups, (2) differences between the two groups at baseline and 8 weeks. If gender differences were observed in the measurements, these measurements were analyzed after stratification by gender. Serum lipid concentrations were analyzed after stratification by hyperlipidemia and non-hyperlipidemia. In all analyses, a two-sided *p*-value < 0.05 was considered significant.

## 5. Conclusions

In conclusion, boiled kelp powder enhanced weight loss and improved body fat percentage in overweight male subjects after 8 weeks of supplementation (3 g/d alginate intake). In addition, boiled kelp powder may reduce serum LDL cholesterol concentrations in non-hyperlipidemic subjects. Additional supplementation of iodine from kelp did not cause serum TSH elevation in Japanese subjects with relatively high intake of iodine from seaweed.

## Figures and Tables

**Table 1:** Baseline characteristics (mean ± SE) of the participants.

		Placebo	Test	*p* ^†^
n		24	24	
	Male	11	12	
	Female	13	12	
Age (y)		47.5 ± 1.9	47.7 ± 2.1	0.96
	Male	44.9 ± 3.4	46.1 ± 3.2	0.80
	Female	49.8 ± 2.0	49.3 ± 2.8	0.88
Body weight (kg)		74.0 ± 1.7	74.4 ± 1.4	0.86
	Male	80.9 ± 1.7	79.0 ± 1.6	0.42
	Female	68.1 ± 1.5	69.8 ± 1.3	0.42
BMI (kg/cm^2^)		27.3 ± 0.3	27.2 ± 1.2	0.79
	Male	27.1 ± 0.3	27.0 ± 0.3	0.90
	Female	27.4 ± 0.5	27.3 ± 0.4	0.85
Body fat percentage (%)		34.3 ± 1.4	32.9 ± 1.3	0.45
	Male	28.5 ± 1.3	27.6 ± 1.1	0.59
	Female	39.2 ± 1.1	38.1 ± 1.0	0.49
Visceral fat area (cm^2^)		82.3 ± 5.7	82.5 ± 5.3	0.99
	Male	92.9 ± 8.2	90.5 ± 7.1	0.83
	Female	73.4 ± 7.2	74.4 ± 7.3	0.92
Systolic blood pressure (mmHg)		122.8 ± 1.9	125.5 ± 2.4	0.38
	Male	125.5 ± 3.3	125.3 ± 3.6	0.97
	Female	120.5 ± 7.8	125.6 ± 3.2	0.20
Diastolic blood pressure (mmHg)		79.1 ± 1.8	81.7 ± 2.0	0.35
	Male	81.0 ± 2.7	81.7 ± 2.9	0.87
	Female	77.5 ± 2.5	81.7 ± 2.9	0.29

^†^*p*-value for the *t*-test of baseline values between the placebo and test groups. No significant differences were observed.

**Table 2:** Daily energy and nutrient intakes during the experimental period (mean ± SE) ^1^.

Week		0	8	8-0 W	*p* (8-0 W) ^†^
Energy (kcal)	Placebo	1922 ± 70	1827 ± 87	−95 ± 52	0.22
	Test	1900 ± 64	1945 ± 121	45 ± 99	
Protein (g)	Placebo	73.4 ± 2.6	70.1 ± 3.8	−3.1 ± 2.8	0.44
	Test	75.9 ± 3.2	76.4 ± 4.4	0.5 ± 3.8	
Fat (g)	Placebo	65.6 ± 3.4	60.4 ± 3.8	−5.1 ± 2.9	0.69
	Test	67.2 ± 3.7	64.0 ± 5.5	−3.1 ± 3.9	
Carbohydrate (g)	Placebo	243.8 ± 11.4	233.7 ± 12.9	−10.1 ± 9.1	0.15
	Test	236.5 ± 10.8	254.2 ± 19.4	17.7 ± 16.5	
Cholesterol (mg)	Placebo	373.3 ± 23.4	345.1 ± 22.0	−27.1 ± 16.6	0.37
	Test	362.4 ± 24.0	363.0 ± 28.6	0.7 ± 26.1	
Total dietary fiber (g)	Placebo	13.6 ± 1.5	11.6 ± 0.9	1.1 ± 3.3	0.84
	Test	13.6 ± 1.2	12.6 ± 1.1	−0.2 ± 5.2	
Calcium (mg)	Placebo	484.8 ± 42.9	495.5 ± 63.8	10.7 ± 42.8	0.99
	Test	523.3 ± 35.8	531.9 ± 56.3	10.1 ± 66.9	
Iodine (μg)	Placebo	548.1 ± 123.4	686.9 ± 176.3	138.8 ± 107.6	0.52
	Test	590.7 ± 150.5	836.6 ± 144.3	254.9 ± 123.6	

^1^ Average daily record for 3 days; significant differences were not observed at 0 and 8 weeks between the placebo and test groups. ^†^ *p*-value for the *t*-test of the 8-0 W values between the placebo and test groups.

**Table 3:** Effects of boiled kelp powder on serum lipids (mean ± SE).

		Placebo (n = 24)		8-0 W	Test (n = 24)		8-0 W	*p* (8-0 W) †
Week		0	8		0	8		
Total cholesterol (mmol/L)	Overall	5.73 ± 0.19	5.61 ± 0.20	−0.12 ± 0.09	5.71 ± 0.19	5.59 ± 0.22	−0.12 ± 0.09	0.987
	HL	6.29 ± 0.25	5.96 ± 0.27	−0.33 ± 0.11	6.33 ± 0.20	6.26 ± 0.28	−0.06 ± 0.13	0.125
	Non-HL	5.07 ± 0.11	5.20 ± 0.17	0.12 ± 0.11	4.98 ± 0.14	4.79 ± 0.09	−0.19 ± 0.14	0.111
LDL cholesterol (mmol/L)	Overall	3.54 ± 0.16	3.45 ± 0.17	−0.09 ± 0.11	3.53 ± 0.18	3.38 ± 0.20	−0.15 ± 0.11	0.689
	HL	3.98 ± 0.21	3.65 ± 0.28	−0.33 ± 0.14	4.08 ± 0.21	3.94 ± 0.28	−0.14 ± 0.17	0.394
	Non-HL	3.02 ± 0.12	3.21 ± 0.15	0.19 ± 0.12	2.88 ± 0.12	2.72 ± 0.10 *	−0.17 ± 0.14	0.063
HDL cholesterol (mmol/L)	Overall	1.49 ± 0.07	1.42 ± 0.07	−0.07 ± 0.02	1.45 ± 0.07	1.42 ± 0.07	−0.02 ± 0.02	0.154
	HL	1.45 ± 0.11	1.40 ± 0.11	−0.05 ± 0.03	1.31 ± 0.08	1.28 ± 0.08	−0.03 ± 0.03	0.675
	Non-HL	1.53 ± 0.09	1.44 ± 0.08	−0.09 ± 0.03	1.60 ± 0.11	1.59 ± 0.11	−0.01 ± 0.04	0.125
Triglyceride (mmol/L)	Overall	1.58 ± 0.14	1.61 ± 0.23	0.03 ± 0.21	1.48 ± 0.23	1.66 ± 0.52	0.18 ± 0.45	0.754
	HL	2.02 ± 0.17	2.15 ± 0.40	0.13 ± 0.40	1.99 ± 0.36	2.34 ± 0.93	0.36 ± 0.83	0.811
	Non-HL	1.06 ± 0.09	0.96 ± 0.10	−0.01 ± 0.10	0.88 ± 0.09	0.86 ± 0.07	−0.02 ± 0.10	0.557

HL: hyperlipidemia (each n = 13), non-HL: non-hyperlipidemia (each n = 11). Mean values were significantly different from the placebo group for the same week: * *p* < 0.05, ^†^ *p*-values for changes after placebo and test diet consumption.

**Table 4:** Changes in thyroid hormone concentrations during the experimental period in subjects (mean ± SE).

	Placebo (n = 24)		8-0 W	Test (n = 24)		8-0 W	*p* (8-0 W) ^†^
Week	0	8		0	8		
TSH (mIU/L)	1.43 ± 0.15	1.43 ± 0.58	−0.01 ± 0.08	1.65 ± 0.39	1.65 ± 0.99	0.01 ± 0.24	0.653
FT3 (pmol/L)	4.24 ± 0.08	4.06 ± 0.10	−0.18 ± 0.07	4.11 ± 0.10	4.05 ± 0.11	−0.06 ± 0.09	0.294
FT4 (pmol/L)	0.12 ± 0.00	0.13 ± 0.00	0.01 ± 0.00	0.12 ± 0.00	0.13 ± 0.00	0.01 ± 0.00	0.826

^†^ *p*-values for changes after placebo and test tablet consumption (Welch’s *t*-tests).

**Table 5:** Test tablet components (g/100 g).

	Placebo	Test
Boiled kelp powder	0	39.3
Maltitol	47.5	24.2
Microcrystalline cellulose	47.5	31.5
Silicon dioxide	1.5	2
Tricalcium phosphate	0	2
Calcium stearate	1.1	1
Coloring	2.1	0
Sodium glutamate	0.2	0
Kelp flavor powder	0.1	0

The weight of one test tablet was 500 mg, and it contained 0.11 g total dietary fiber, 34.3 μg iodine, and 109.3 mg alginate.

## Data Availability

Data is contained within the article or supplementary material.

## Supplementary Materials

The following are available online at https://www.mdpi.com/article/10.3390/md19070352/s1, Table S1: Effects of boiled kelp powder on body weight, BMI, body fat percentage, visceral fat area, and blood pressure (mean ± SE).

## Abbreviations

BMI	body mass index
HDL	high-density lipoprotein
ITT	intention to treat
LDL	low-density lipoprotein
MS	metabolic syndrome
PP	per-protocol
SE	standard error of the mean
