# Supplementary material for: Effects of Daily Kelp (Laminaria japonica) Intake on Body Composition, Serum Lipid Levels, and Thyroid Hormone Levels in Healthy Japanese Adults: A Randomized, Double-Blind Study
Source: Mar Drugs. 2021 Jun 22;19(7):352. doi: 10.3390/md19070352 (PMC8307228; doi:10.3390/md19070352)
Supplement: Supplementary file 1 [file marinedrugs-19-00352-s001.zip › marinedrugs-1247183-supplementary.pdf]

Table S1. Effects of boiled kelp powder on body weight, BMI, body fat percentage, visceral fat area, and blood pressure (mean±SE)

		Placebo(n=24)		8-0W	Test(n=24)		8-0W	p(8-0W)†
		0	8		0	8		
Body weight(kg)	male	80.9±1.8	81.3±1.8	0.39±0.48	79.0±1.0	78.1±5.3	-0.89±0.46	0.065
	Female	68.1±1.6	68.1±1.6	0.01±0.40	69.8±1.3	70.0±1.2	0.24±0.35	0.665
BMI (kg/cm^2)	male	27.1±0.4	27.0±0.4	0.13±0.12	27.0±0.3	26.7±0.3	-0.30±0.19	0.072
	Female	27.4±0.5	27.4±0.4	-0.01±0.16	27.3±0.40	27.4±0.4	0.11±0.13	0.587
Body fat percentage (%)	male	28.5±1.3	28.5±1.3	-0.05±0.22	27.6±1.1	26.8±3.5	-0.84±0.35	0.049
	Female	39.2±1.1	38.5±1.1	-0.66±0.39	38.1±1.0	37.3±1.1	-0.72±0.40	0.992
Visceral fat area (cm^2)	male	92.9±8.2	90.5±6.2	-2.36±3.88	90.5±7.1	84.8±7.3	-5.75±5.72	0.630
	Female	73.4±7.2	71.5±6.1	-1.90±5.10	74.4±7.3	72.4±8.5	-2.00±3.90	0.991
Systolic blood pressure (mmHg)	male	125.5±3.3	121.7±4.3	-3.4±3.3	125.3±3.6	121.1±3.1	-4.2±2.9	0.921
	Female	120.5±7.8	119.8±2.5	-0.6±2.0	125.6±3.2	123.6±3.5	-2.1±2.3	0.636
Diastolic blood pressure (mmHg)	male	81.0±2.4	75.9±3.6	-5.1±2.4	81.7±2.9	74.8±1.8	-6.9±1.5	0.777
	Female	77.5±2.5	75.7±2.5	-1.8±2.2	81.7±2.9	76.6±3.2	-5.1±1.8	0.829

†p values for changes after placebo and test consumption (Welch's t-test or Wilcoxon rank sum test).
